# Analysis of the outcome and costs of lower back pain should not only rely on electronic data but also use real-world experiences

**DOI:** 10.1590/1516-3180.2025.3106.11112025

**Published:** 2026-03-27

**Authors:** Josef Finsterer

**Affiliations:** I Professor, Neurology Department, Neurology and Neurophysiology Center, Vienna, Austria.

 Dear Editors, 

 We enjoyed reading the article by Zanuto et al.^1^ on the costs, classification, and effects of physical activity on lower back pain in 198 patients analyzed using the Nordic and Baecke questionnaire at baseline and 6, 12, and 18 months later. Lower back pain was associated with female sex and young age and incurred high costs for medical consultations, while cycling was much cheaper. Although this study is noteworthy, several points must be discussed. 

 First, the data were partly extracted from the electronic medical records of the included patients. However, electronic data have the disadvantage that data may be missing, the extracted data may be inconsistent, it is not easy to check the accuracy of the data, and desirable new data can no longer be generated. It is important to know how many patients had to be excluded because of missing or incorrect data. 

 The second issue is the discrepancy between the aims of the study (analyzing the cost of chronic lower back pain and its correlates in adults during an 18-month follow-up period) and the assessment of musculoskeletal symptoms such as pain, formication, or numbness in the neck, shoulder, upper back, elbows, and wrists/hands. These body regions cannot be considered to be related to the lower back; therefore, patients with symptoms related to the upper limbs, neck, and upper back should be excluded from the analysis. The inclusion of this data may massively bias the results, especially in cases in which no other symptoms in the lower spine have been reported in addition to these symptoms. Patients with these types of symptoms should be excluded from the study. 

 Third, the cost of diagnostic and therapeutic management was calculated by reviewing the demand for services in medical records. However, it is possible that patients obtain their analgesic medication via the Internet or the black market. In addition, many patients resort to alternative treatments, which are also not recorded in the medical records. Therefore, their costs do not appear in the medical records, suggesting that the true cost of treating chronic lower back pain is much higher. If lower back pain is a psychosomatic manifestation of depression and is treated with psychotherapy, these costs will also not appear in the total costs. 

 Fourth, it was unclear why television viewing was included in the analysis of physical activity. Watching television usually takes place while sitting or lying down and is hardly associated with physical activity. Therefore, this item should be excluded from the Baecke questionnaire. 

 Fifth, the actual cause of the lower back pain was not included in the analysis. Lower back pain due to plasmacytoma, bone metastases, or spinal cord stroke may lead to results different from those of lower back pain due to viral infection, stress, or Elsberg’s syndrome. Because the study results are highly dependent on the underlying cause of the lower back pain, the etiology should be considered in the analysis. 

 In summary, lower back pain should not be confused with neck pain, and the cause of lower back pain should be included in analyses when performing longitudinal studies. 
